# Supplementary material for: Rational Design of Multiclade Coronavirus Spike Immunodominant Domain Nanoparticles to Elicit Broad Antibody Responses
Source: bioRxiv. 2025 Oct 1:2025.10.01.679649. Preprint. [Version 1] doi: 10.1101/2025.10.01.679649 (PMC12621691; doi:10.1101/2025.10.01.679649)
Supplement: Supplement 2 [file media-2.pdf]

Rational Design of Multiclade Coronavirus Spike Immunodominant Domain Nanoparticles Elicit Broad Antibody Responses

Christian K.O. Dzuvor[1], Sydney Moak[1,2], Lindsay R. McManus[1], Abigail Thomas[1,3], Abigail E. Dzordzorme[1,4,6], Taewoo Kim[1], Jeswin Joseph[1],b Valerie Foley[1,3], Laura Novik[5], Ingelise J. Gordon[5], LaSonji A. Holman[5], Lesia K. Dropulic[5], Ryan P. McNamara[1], Kizzmekia S. Corbett-Helaire[1,2,7]

[1]Department of Immunology and Infectious Diseases; Harvard T.H. Chan School of Public Health; Boston, Massachusetts, 02115; United States of America

[2]Howard Hughes Medical Institute; Chevy Chase, Maryland, 20815; United States of America

[3]Northeastern University, Boston, Massachusetts, 02115; United States of America

[4]Harvard-MIT Division of Health Sciences and Technology, Institute for Medical Engineering and Science, Massachusetts Institute of Technology, Cambridge, Massachusetts, 02139; United States of America

[5]Vaccine Research Center; National Institutes of Allergy and Infectious Diseases; National Institutes of Health; Bethesda, Maryland, 20892; United States of America

[6]Current Affiliation: Wyss Institute for Biologically Inspired Engineering, Harvard University, Boston, Massachusetts,02215; United States of America

[7]Correspondance: kizzmekia_corbett@hsph.harvard.edu

Supplementary Figures

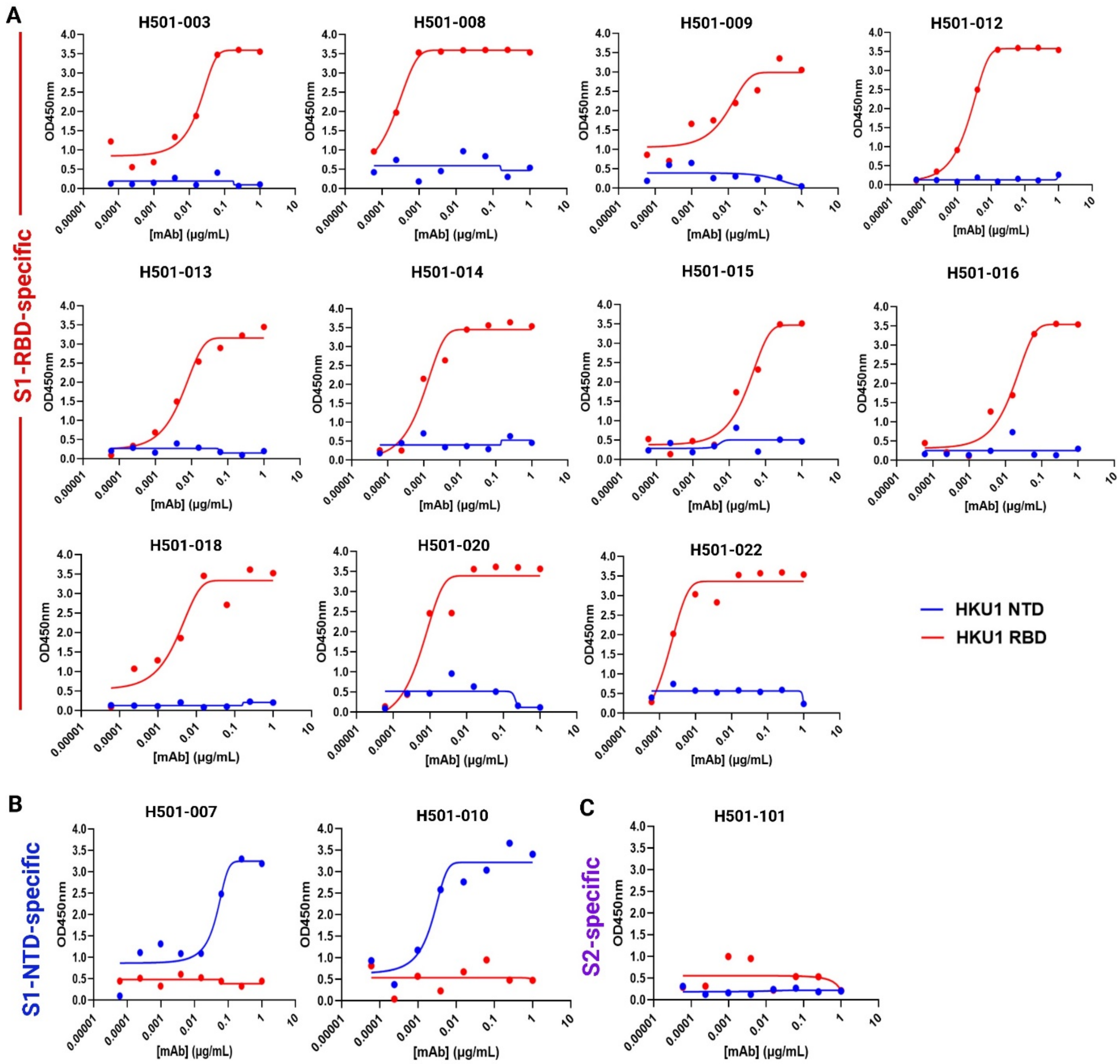

Supplementary Fig. 1: Antigenic characterization of HCoV-HKU1 S1 domain probes. (**A**) S1-RBD-specific mAbs, (**B**) S1-NTD-specific mAbs and (**C**) S2-specific mAb isolated from convalescent HCoV-HKU1-positive PBMCs were screened by ELISA for binding to HKU1 RBD (red) and HKU1 NTD (Blue) probes.

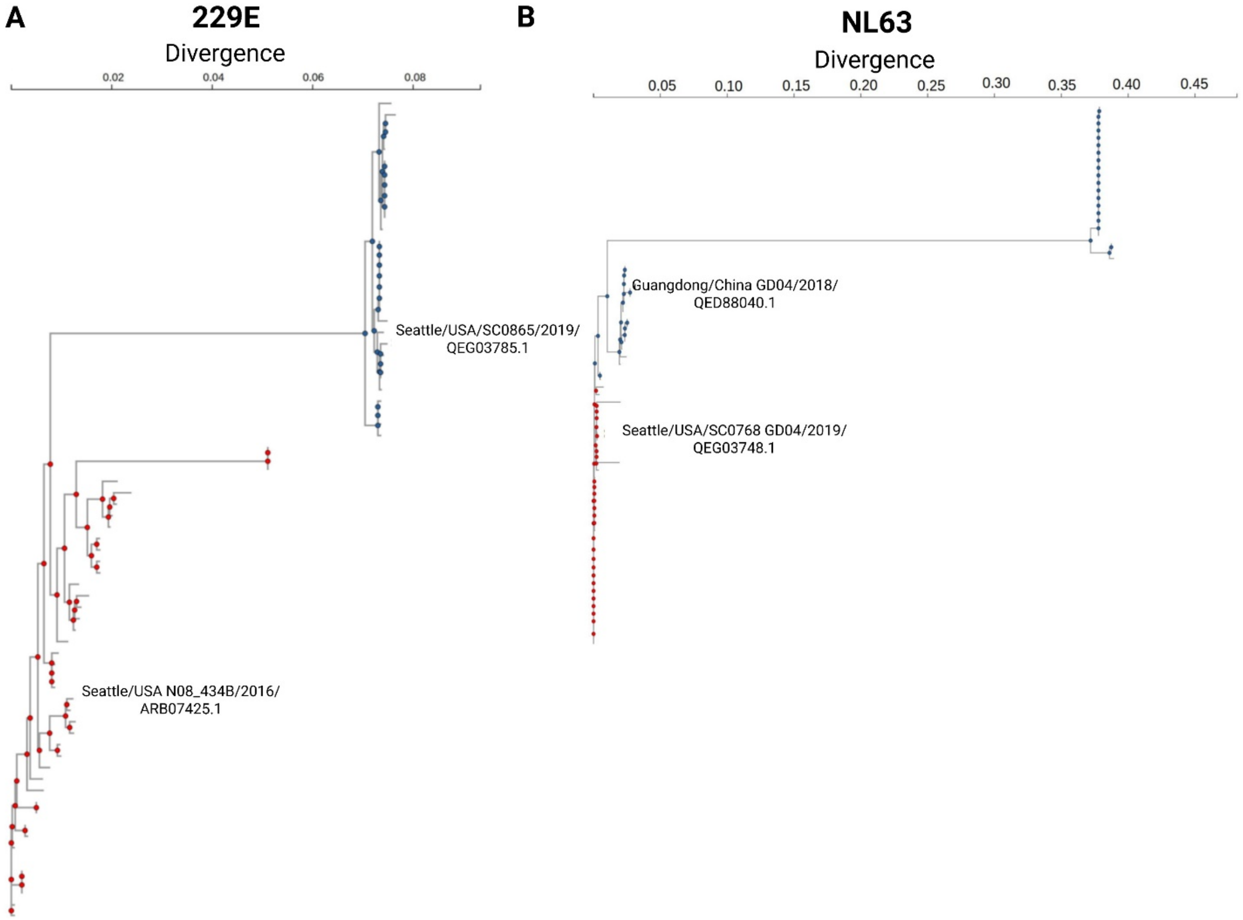
A
229E
Divergence
0.02
0.04
0.06
0.08
Seattle/USA/SC0865/2019/
QEG03785.1
Seattle/USA N08_434B/2016/
ARB07425.1
B
NL63
Divergence
0.05
0.10
0.15
0.20
0.25
0.30
0.35
0.40
0.45
Guangdong/China GD04/2018/
QED88040.1
Seattle/USA/SC0768 GD04/2019/
QEG03748.1

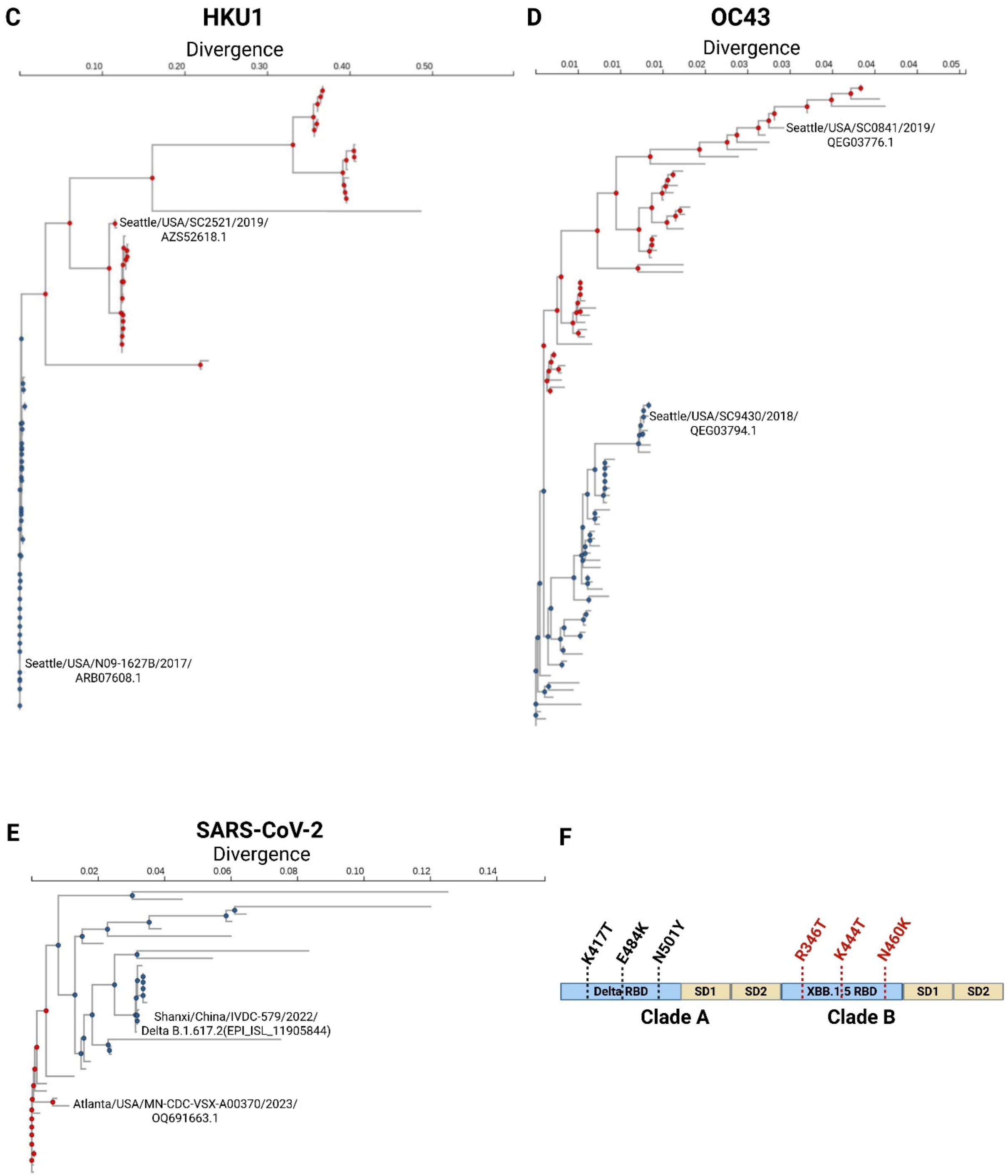

Supplementary Fig. 2: Selection of EhCoV immunodominant domain (IDD) vaccine antigens, related to Fig. 1H. Spike Protein Sequences were identified using Nextstrain database and filtered by timeframe. (**A-E**) Phylogenetic trees were created with Clustal Omega EMBL-EBI using seeded guide trees and HMM profile-profile techniques and aligned by Clustal Omega MSA for EhCoV (**A**) 229E, (**B**) NL63 (**C**) HKU1, (**D**) OC43 and (**E**) SARS-CoV-2. Clades A and B are highlighted in blue and red, respectively. Selected S

proteins for IDD vaccine designs are indicated. (**F**) Schematic depiction of mutation patches used to expand SARS-CoV-2 cross-variant antigenic coverage on the SARS-CoV-2 IDD construct.

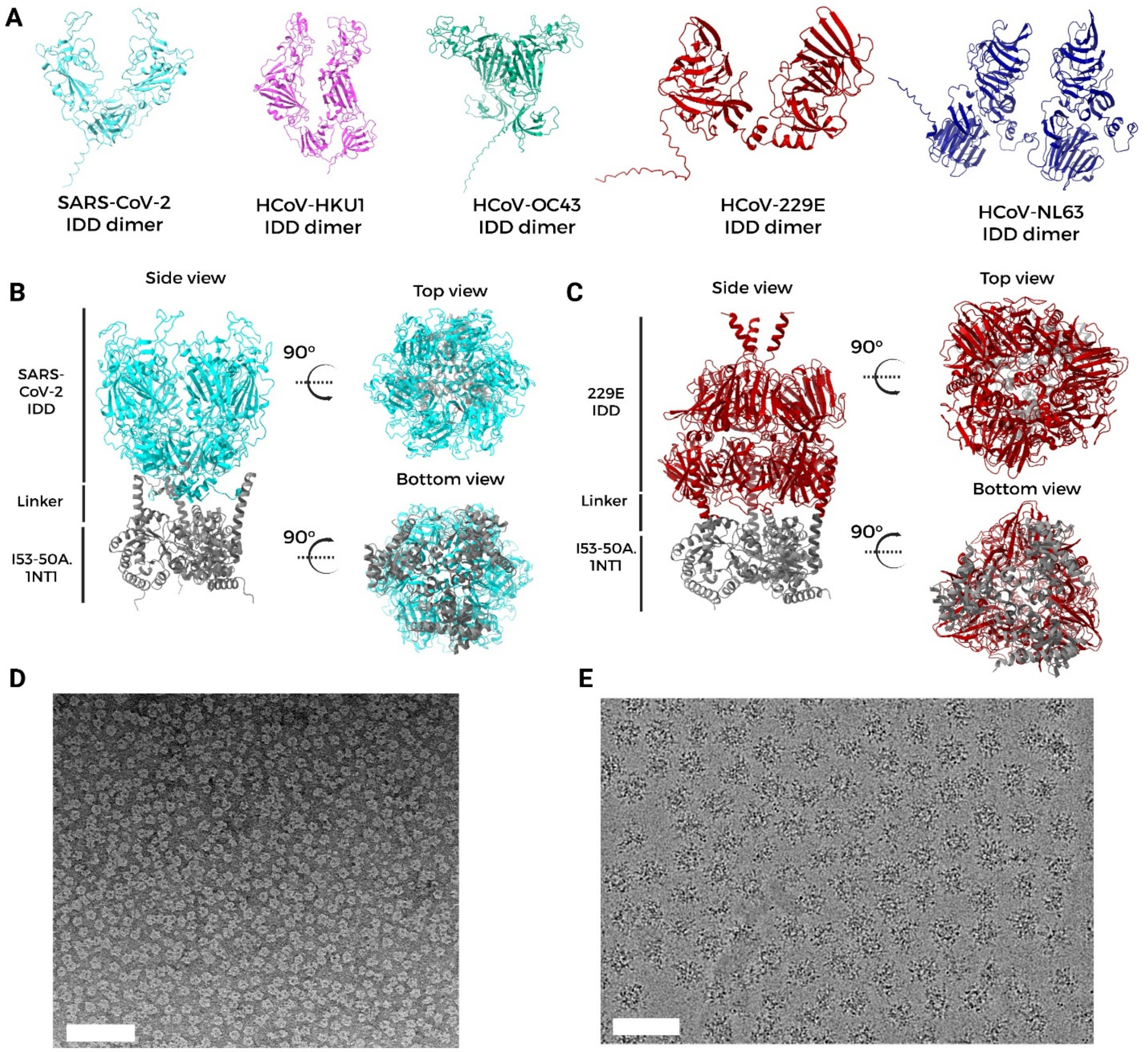

Supplementary Fig. 3: IDD design and NP characterization, related to Fig. 2. (**A**) AlphaFold structure prediction of monomeric chimeric IDD antigens. Molecular models of five EhCoV IDDs. (**B-C**) Composite molecular models of chimeric immunodominant domain (IDD) trimers for representative endemic (**B**) β-hCoV (SARS-CoV-2) and (**C**) α-hCoV (229E). The C-terminus of SARS-CoV-2 IDD (cyan) and 229E-CoV IDD (red) were fused to the N-termini of I53-50A trimer (gray) via a linker. (**D**) Representative negative-stain EM of purified bare I53-50 NP. Scale bar, 100 nm. (**E**) Representative cryo-EM micrograph of mosaic-5 IDD NP embedded in vitreous ice. Scale bar, 50 nm.

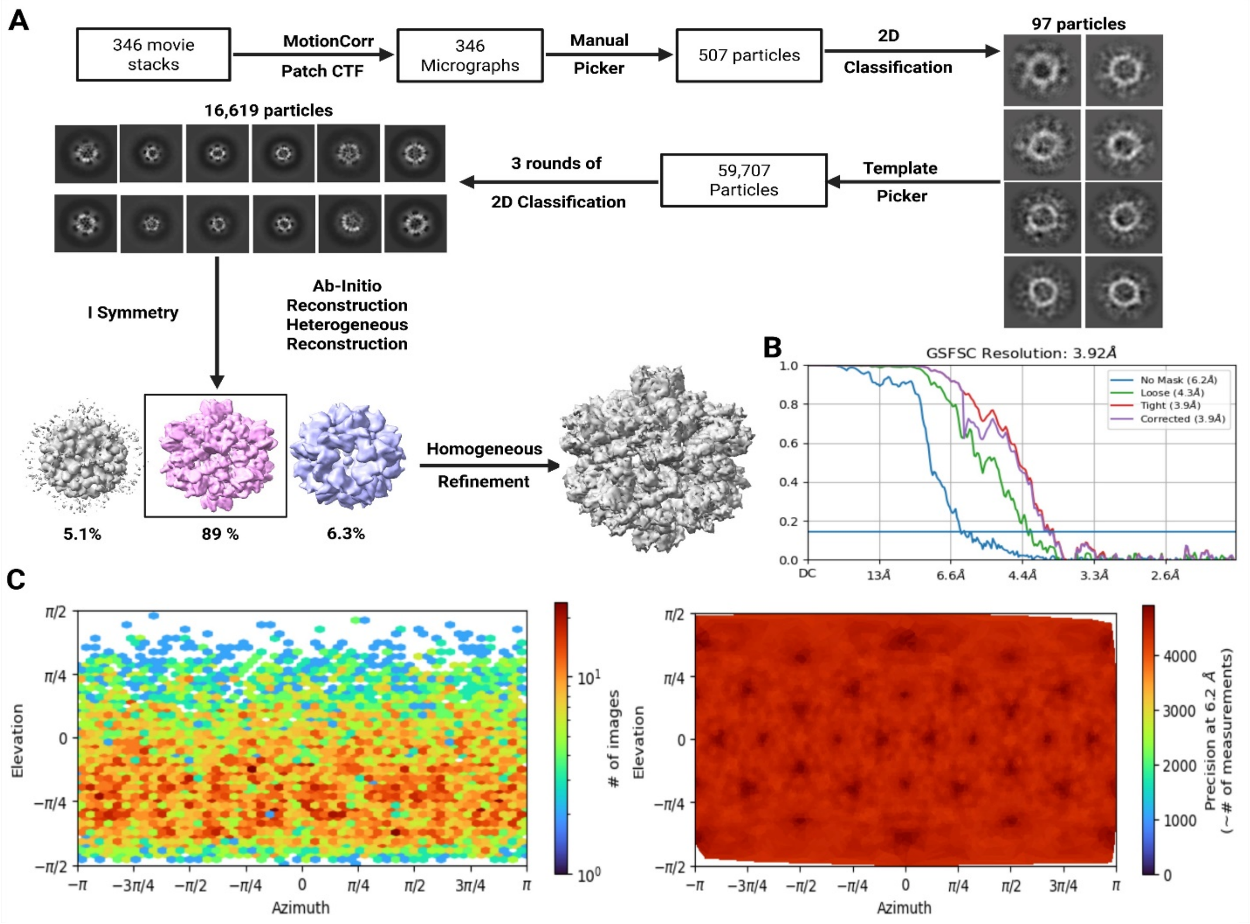

Supplementary Fig. 4: Cryo-EM structure determination of Mosaic-5 IDD NP, related to Figure 2D-E. (**A**) Cryo-EM data processing and reconstruction workflow showcasing all relevant and standard steps. 3D reconstruction was done using icosahedral symmetry. Final resolution was 3.9Å. (**B**) Gold-standard Fourier shell correlation (FSC) curve for the Mosaic-5 IDD NP density map shown in **Fig. 2E**. The 0.143 cut-off value is indicated by horizontal blue bar. (**C**) 2D Euler angular distribution plots used in reconstructions.

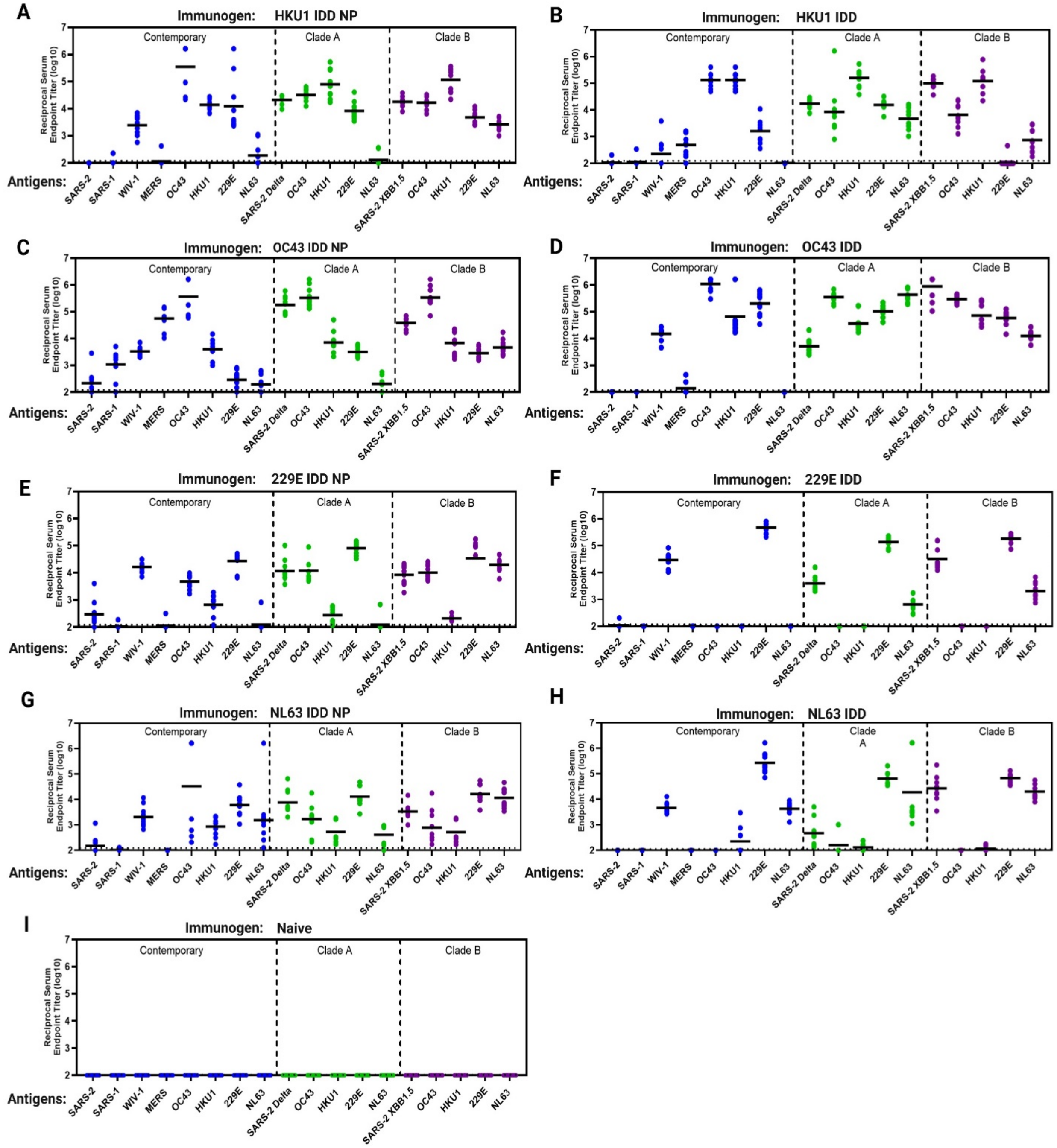

Supplementary Fig. 5: (A-I) Cross-reactive antibody responses in EhCoV IDD and IDD NP immunized mice, related to Fig. 3. Female BALB/cJ mice (N = 10/group) were immunized at weeks 0 and 3 with 5 ug of IDD trimer (**A, C, E, G**) or IDD NPs (**B, D, F, H**) or Naive (**I**) with SAS adjuvant and bled at week 5 for serology. Control mice were immunized with adjuvanted PBS. Immune sera were assessed for antibody binding to various CoV S proteins by ELISA. Contemporary CoV S proteins are shown in blue. Clade A CoV S proteins are shown in green. Clade B CoV S proteins are shown in purple. Each dot represents an individual mouse. Horizontal dashed lines represent assay LOD.

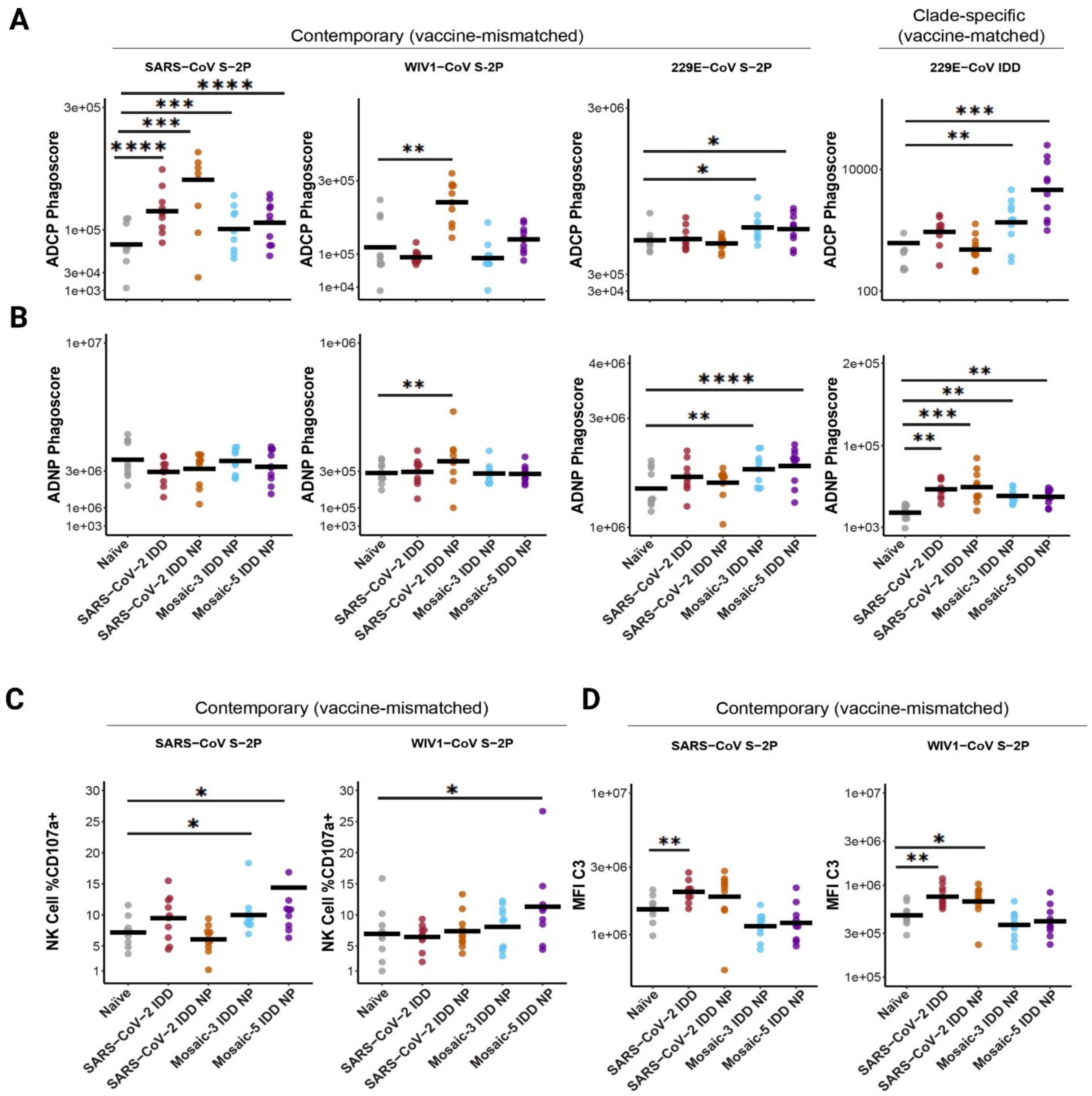

Supplementary Fig. 6: Antibody Fc-dependent functional scores (ADCP, ADNP, ADNK, ADCD) against pandemic threat and endemic coronavirus(229E-CoV). (**A, B**) Fc-mediated effector functions of the naïve and immune sera after vaccination with IDD NPs against contemporary (SARS-CoV S-2P, WIV1-CoV S-2P, and 229E-CoV S-2P) and clade-specific (229E-CoV IDD) protein. Antibody-mediated cellular phagocytosis with monocytes (ADCP, **A**) or neutrophils (ADNP, **B**) using IDD-vaccine-induced immune and naïve sera and beads coated with indicated SARS-CoV, WIV1-CoV, and 229E-CoV proteins. (**C**) Antibody-dependent natural killer cell activation (ADNKA) using IDD-vaccine-induced immune and naïve sera and beads coated with indicated SARS-CoV and WIV1-CoV S-2P proteins, as measured by the percentages of NK cells expressing CD107a against the antigens by vaccine group indicated. (**D**) Antibody-dependent complement deposition (ADCD) on beads coated with indicated SARS-CoV and WIV1-CoV S-2P proteins, and incubated with naïve or immune sera. In all figures, the horizontal black bar indicates the

group mean value (N=10 mice per group). Each dot represents an individual mouse in a group. Two–way ANOVA with Tukey's post-hoc test was performed. The statistical significance is displayed as follows: ∗p < 0.05; ∗∗p < 0.01; ∗∗∗p < 0.001; ∗∗∗∗p < 0.0001. Source data file is provided.

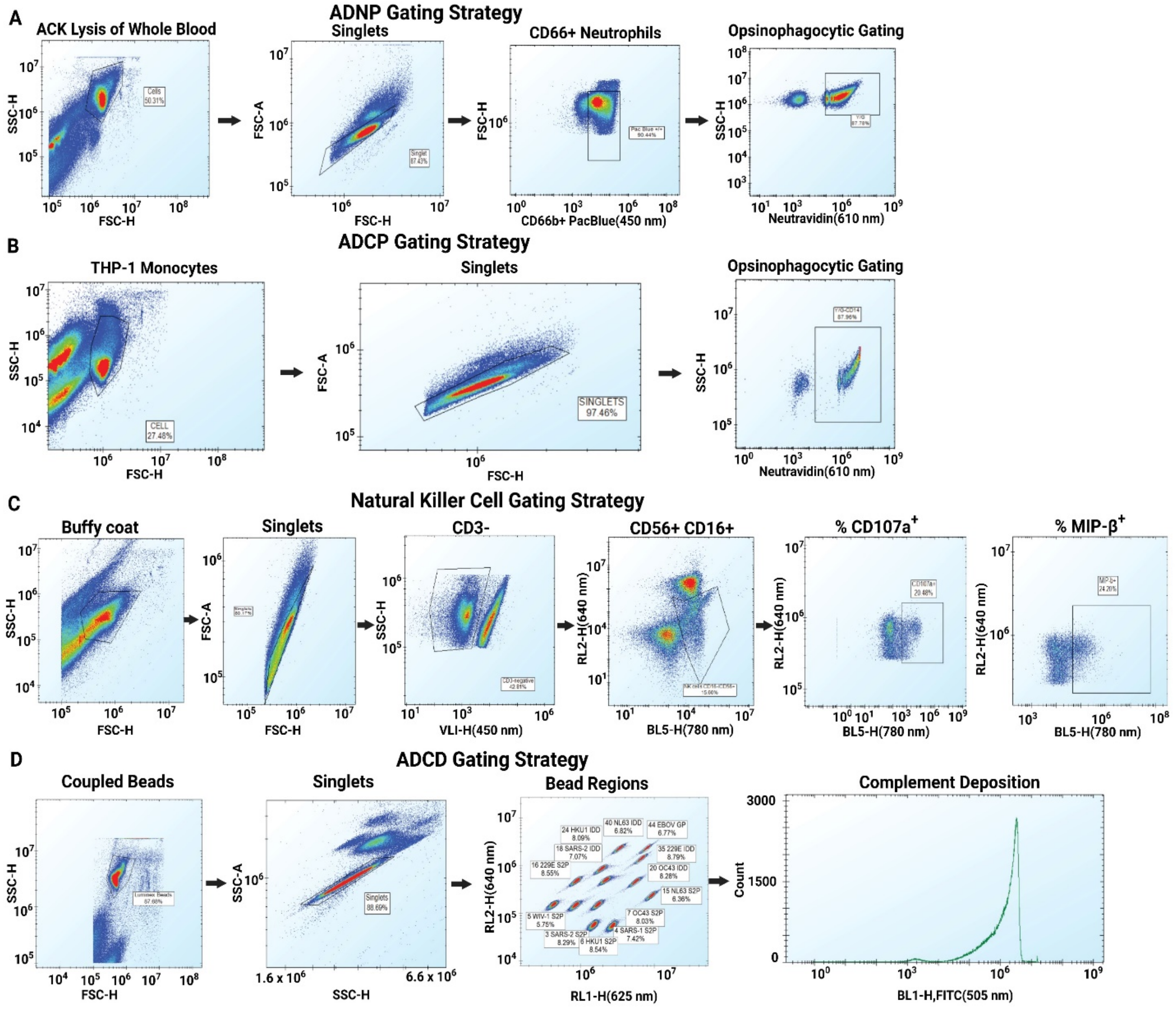

Supplementary Fig. 7: Flow cytometry gating strategy for Fc effector function assays, related to Fig. 5, Extended data Fig. 7 and Supplementary Fig. 6. (A) Gating for antibody-dependent neutrophil phagocytosis (ADNP) assay displaying CD66+ neutrophils with phagocytosed beads. (**B**) Gating for antibody-dependent cellular phagocytosis by monocytes (ADCP) assay displaying CD14+ monocytes with phagocytosed beads. (**C**) Gating for antibody-dependent natural killer cell activation (ADNKA) displaying CD56+/CD16-, and CD107a expression. (**D**) Gating for antibody-dependent complement deposition (ADCD) on CoV protein and antibody-coated beads.

Supplementary Tables

Supplementary Table. 1. EhCoV convalescent donor information and sample details

VRC Study	Subject ID	Date of Confirmed CoV Infection	Sample Type	Blood Draw Information	
				Date	Months after CoV[1]
VRC 200	A	1/30/2014	PBMC	3/12/2014	1
VRC 500			Serum	2/10/2015	13
VRC 317	B	5/3/2018	Serum	7/11/2018	2
			PBMC		
	C	7/12/2018	Serum	10/4/2018	3
			PMBC		
	D	1/10/2018	Seum	4/12/2018	3
			PBMC		
VRC 200	E	3/20/2020	Serum	6/24/2020	3
			PBMC		

[1]Date of blood draw – Date of confirmed CoV infection)/12 = Months after CoV

Supplementary Table .2. Cryo-EM Data collection and processing parameters, related to Figure 2D-E.

Cryo-EM Data Collection and Processing Statistics	
**EM Data Collection**	
Microscope	Talos Arctica
Software	SerialEM
Voltage(kV)	200
Detector	Gatan K3
Magnification(Norminal)	36,000
Electron exposure(e-/ Å^2)	60.04
Defocus range (μm)	0.1-2.4
Pixel size(Å)	1.1
Flux (e-/pix/ Å)	12
Exposure time(seconds)	4.224
Total frames	47
Micrographs collected	346

3D Reconstruction Statistics	
Software	CryoSPARC v4.6.2
Symmetry imposed	I
Box size(pix)	450
Initial particle image no	346
Final particle image no	170
Map sharpening B-factor	-134
FSC threshold	0.143
Unmasked resolution at 0.143FSC(Å)	6.2
Masked resolution at 0.143FSC(Å)	3.9
Map resolution(Å)	3.92

Supplementary Table. 3: Sequences of designed immunogens and expression constructs reported in this study.

**Construct Name**	**Amino Acid Sequence**
**Color Key**	Secretion Signal Sequence, Clade A domain, Clade B domain, GS linker, Display Linker, I53-50A.1NT1, Streptag II, His-8-tag, I53-50B.4PT1
**SARS-CoV-2 IDD-Linker-I53-50A.1NT1**	MRGLGTCLATLAGLLLTAAGRFPNITNLCPFGEVFNATRFASVYAWNRKRISNCVADYSVLYNSASFSTFKCYGVSPTKLNDLCFTNVY<br>ADSFVIRGDEVRQIAPGQTGTIADYNYKLPDDFTGCVIAWNSNNLDSKVGGNYNYRYRLFRKSNLKPFERDISTEIYQAGSKPCNGVK<br>GFNCYF PLQSYGFQPT YGVGYQPYRV VVLSFELLHA PATVCGPKKS TN LVKNKCVN FNFNGLTGTG VLTESNKKFL PFQQFGRDIA<br>DTTDAVRDPQTLEILDITPCSFGGGGSRFPNITNLCPFHEVFNATTFASVYAWNRKRISNCVADYSVIYNFAPFFAFKCYGVSPTKLNDLC<br>FTNVYADSFVIRGNEVSQIAPGQTGNIADYNYKLPDDFTGCVIAWNSNKLDSTPSGNYNYRYRLFRKSKLKPFERDISTEIYQAGNKPC<br>NGVA GPNCYSPLQS YGFRPTYGVG HQPYRVVVLS FELLHAPATV CGPKKSTNLVKNKCVNFNFNGLTGTGVLTE SNKKFLPFQQ<br>FGRDIADTTDAVRDPQTLEILDITPCSFGGGGGGSAEAAAKASSAEAAAKEAAAKEAAAKEEAARKMEELFKKHKIVAVLRANSVEEA<br>IEKAVAVFAGGVHLIEITFTVPDADTVIKALSVLKEKGAIIGAGTVTSVEQCRKAVESGAEFIVSPHLDEEISQFCKEKGVFYMPGVMTPT<br>ELVKAMKLGHDILKLFPGEVVGPEFVKAMKGPFPNVKFVPTGGVDLDNVCEWFDAGVLAVGVGDALVEGDPDEVREKAKEFVEKIR<br>GCTEGSLEWSHPQFEKGSGHHHHHHHH
**HCoV-HKU1 IDD-Linker-I53-50A.1NT1**	MDAMKRGLCCVLLLCGAVFVSPSASGSTVKPVATVHRRIPDLPDCDID KWLNNFNVPS PLNWERKIFS NCNFNLSTLL RLVHTDSFSC<br>NNFDESKIYGSCFKSIVLDK FAIPNSRRSD LQLGSSGFLQ SSNYKIDTTS SSCQLYYSLP AINVTINNYN PSSWNRRYGF NNFNLSSHSV<br>VYSRYCFSVNNTFCPCAKPSFASSCKSHKPPSASCPIGTNYRSCESTTVLDHTDWCRCSCLPDPITAYDPRSCSQKKSLVGVGEHCAGFG<br>VDEEKCGVLDGSYNVSCLCSTDAFLGWSYDTCVSNNRCNIFSNFILNGINSGTTCSNDLLQPNTEVYTDVCVDYDLYGITGQGIFKEVS<br>AVYYNSWQNLLYDSNGNIIGFKDFVTNKTYNIFPCYAGGGSTVKPVATVYRRIPNLPDCDIDNWLNNVSVPSPLNWERRIFSNCNFNLS<br>TLLRLVHVDSFSCNNLDKSKIFGSCFNSITVDKFAIPNRRRDDLQLGSSGFLQSSNYKIDTTSSSCQLYYSLPAINVTINNYNPSSWNRRY<br>GFNNFNLSSHSVVYSRYCFSVNNTFCPCAKPSFASSCKSHKPPSASCPIGTKYRHCDLDTTLYVKNWCRCSCLPDPISTYSPNTCPQKKV<br>VVGIGEHCPGLGINEEKCGTQLNHSSCSCSPDAFLGWSFDSCISNNRCNIFSNFIFNGINSGTTCSNDLLYSNTDVSTGVCVNYDLYGITG<br>QGIFKEVSAAYYNDWQNLLYDSNGNIIGFKDFLTNKTYTILPCYSGRVSGGGGSAEAAAKASSAEAAAKEAAAKEAAAKEEAARKME<br>ELFKKHKIVAVLRANSVEEAIEKAVAVFAGGVHLIEITFTVPDADTVIKALSVLKEKGAIIGAGTVTSVEQCRKAVESGAEFIVSPHLDEEI<br>SQFCKEKGVFYMPGVMTPTELVKAMKLGHDILKLFPGEVVGPEFVKAMKGPFPNVKFVPTGGVDLDNVCEWFDAGVLAVGVGDAL<br>VEGDPDEVREKAKEFVEKIRGCTEGSLEWSHPQFEKGSGHHHHHHHH
**HCOV-OC43 IDD-Linker-I53-50A.1NT1**	MPMGSLQPLATLYLLGMLVASVLAIADVYRRKPDLPNCNIEAWLNDKSVPSPLNWERKTFSNCNFNMSSLMSFIQADSFTCNNIDAA<br>KIYGMCFSSITIDKFAIPNRRKVDLQLGNLGYLQSSNYRIDTTATSCQLYYNLPAANVSVSRFNPSTWNKRFGFIEDAVFKPQPAGVLT<br>NHDVVYAQHCFKAPKNFCPCSSCSGKNNGIGTCPAGTNSLTCDNLCTLDPITLKAPDTYKCPQSKSLVGIGEHCSGLAVKSDYCGNNS<br>CTCQPQAFLGWSADSCLQGDKCNIFANFILHDVNNGLTCSTDLQKANTEIELGVCVNYDLYGISGQGIFVEVNATYYNSWQNLLYDS<br>NGNLYGFRDYITNRTFMIHSCYSGGGSGVYELNGYTVQPIADVYRRKPDLPNCNIEAWLNDKSVPSPLNWERKTFSNCNFNMSSLMS

	FIQADSFTCNNIDAAKIYGMCFSSITIDKFAIPNGRKVDLQLGNLGYLQSFNYRIDTTATSCQLYYNLPAANVSVSRFNPSTWNKRFGFI<br>EDSVFKPQPAGVLTDHDVVYAQHCFKAPKNFCPCKSNSSLCVGSGPGKNNGIGTCPAGTNYLTCHNLCNPDPITFTGPYKCPQTKSLV<br>GIGEHCSGLAVKSDYCGGNPCTCQPQAFLGWSADSCLQGDKCNIFANLILHDVNSGLTCSTDLQKANTDIKLGVCVNYDLYGISGQGI<br>FVEVNATYYNSWQNLLYDSNGNLYGFRDYITNRTFMIRSCYSGGGGSAEAAAKASSAEAAAKEAAAKEAAAKEEAARKMEELFKK<br>HKIVAVLRANSVEEAIEKAVAVFAGGVHLIEITFTVPDADTVIKALSVLKEKGAIIGAGTVTSVEQCRKAVESGAEFIVSPHLDEEISQFC<br>KEKGVFYMPGVMTPTELVKAMKLGHDILKLFPGEVVGPEFVKAMKGPFPNVKFVPTGGVDLDNVCEWFDAGVLAVGVGDALVEG<br>DPDEVREKAKEFVEKIRGCTEGSLEWSHPQFEKGSGHHHHHHHH
**HCoV-229E IDD -Linker-I53-50A.1NT1**	MFVLLVAYALLHIAGCQTTNGTNTSHAVCNGCVGHSENVFAVESGGYIPSNFAFNNWFLLTNTSSVVDGVVRSFQPLLLNCLWSVSGSQ<br>VITGFVYFNGTGRGACKGFYSNASSDVIRYNINFEENLRRGTILFKTSYGAVVFYCTNNTLVSGDAHIPSGTVLGNFYCFVNTTIGNETT<br>SAFVGALPKTVREFVISRTGHFYINGYRYFSLGNVEAVNFNVTNAATTVCTVALASYADVLVNVSQTAIANIIYCNSVINRLRCDQLSGG<br>SQTTNGMNTSHSVCNGCVGHSENVFAVESGGYIPSNFAFNNWFLLTNTSSVVDGVVRSFQPLLLNCLWSVSGSRLTTGFVYFNGTGRG<br>DCKGFYSNASSDVIRYNINFEENLRRGTILFKTSYGAVVFYCTNNTLVSGDAHIPSGTVLGNFYCFVNTTIGNETTSAFVGALPKTVREF<br>VISRTGHFYINGYRYFSLGNVEAVNFNVTNAATTVCTVALASYADVLVNVSQTAIANIIYCNSVINRLRCDQLSGGGGSAEAAAKASSA<br>EAAAKEAAAKEAAAKEEAARKMEELFKKHKIVAVLRANSVEEAIEKAVAVFAGGVHLIEITFTVPDADTVIKALSVLKEKGAIIGAGTV<br>TSVEQCRKAVESGAEFIVSPHLDEEISQFCKEKGVFYMPGVMTPTELVKAMKLGHDILKLFPGEVVGPEFVKAMKGPFPNVKFVPTGG<br>VDLDNVCEWFDAGVLAVGVGDALVEG DPDEVREKAKEFVEKIRGCTEGSLEWSHPQFEKGSGHHHHHHHH
**HCoV-NL63 IDD-Linker-I53-50A.1NT1**	MKLFLILLILPLVSCFSTCNSNASISMLQLGVPDNSSTIVTGLLPVHWICANQSTSTYPANGFFYIDVGKHRSAFALHSG YYDANQYYIY<br>LTNNISLNAPVTLKICKFGNTSFACLSNVSTSHHCIVNSSFTEQLGVPLGITISGETVRLHLYNATRTFYVPAAYKLTKLSVKCYFSESC<br>VFSVVNATVTVNVTTLNGRIVNYTVCDDCNGYTDNIFSVQQDGRIPNGFSFNNWFLLTNGSTLVDGVSRLYQPLRLTCLWPVPGLKSS<br>TGFVYFNATGSDVNCNGYQHHSVADVMRYNLNFSSNSVDNLKSGVIVFKTLQYDVLFYCSNSSSGVLDTTIPFGPSSQPYYCFINSTIN<br>TTHVSTFVGVLPPIVREIVVARTGQFYINGFKYFDLGFIEAVNFNVTTASATDFWTVAFATFVDVLVNVSATNIQNLLYCDSPFEKLQC<br>EHLQFGLQDGFYSANFLDDNVLPDGGSSNANLSMLQLGVPDNSSTIVTGLLPTHWICANQSTSVYSANGFFFIDVGNHRSAFALHTG<br>YYDVNQYYIYVTNEIGLNASVTLKICKFGNTTFDFLSNSSSSFDCIVNLLFTEQLGAPLGITISGETVRLHLYNVTRTFYVPAAYKLTKL<br>SVKCYFNYSCVFSVVNATVTVNVTTHNGRVVNYTVCDDCNGYTDNIFSVQQDGRIPNGFPFNNWFLLTNGSTLVDGVSRLYQPLRLT<br>CLWPVPGLKSSTGFVYFNATGSDVNCNGYQHNSVADVMRYNLNFSANSVDNLKSGVIVFKTLQYDVLFYCSNSSSGVLDTTIPFGPSS<br>QPYYCFINSTINTTHVSTFVGVLPPTVREIVVARTGQFYINGFKYFDLGFIEAVNFNVTTASATDFWTVAFATFVDVLVNVSATKIQNLL<br>YCDSPFEKLQCEHLQFGLQDGFYSANFLDDNGGGGSAEAAAKASSAEAAAKEAAAKEAAAKEEAARKMEELFKKHKIVAVLRANS<br>VEEAIEKAVAVFAGGVHLIEITFTVPDADTVIKALSVLKEKGAIIGAGTVTSVEQCRKAVESGAEFIVSPHLDEEISQFCKEKGVFYMPG<br>VMTPTELVKAMKLGHDILKLFPGEVVGPEFVKAMKGPFPNVKFVPTGGVDLDNVCEWFDAGVLAVGVGDALVEGDPDEVREKAKE<br>FVEKIRGCTEGSLEWSHPQFEKGSGHHHHHHHH
**I53-50A.1NT1**	MKMEELFKKHKIVAVLRANSVEEAIEKAVAVFAGGVHLIEITFTVPDADTVIKALSVLKEKGAIIGAGTVTSVEQCRKAVESGAEFIVS<br>PHLDEEISQFCKEKGVFYMPGVMTPTELVKAMKLGHDILKLFPGEVVGPEFVKAMKGPFPNVKFVPTGGVDLDNVCEWFDAGVLAV<br>GVGDALVEG DPDEVREKAKEFVEKIRGCTEGSLEHHHHHHHH
**I53-50B.4PT1**	MNQHSHKDHETVRIAVVRARWHAEIVDACVSAFEAAMRDIGGDRFAVDVFDVPGAYEIPLHARTLAETGRYGAVLGTAFVVNGGIY<br>RHEFVASAVINGMMNVQLNTGVPVLSAVLTPHNYDKSKAHTLLFLALFAVKGMEAARACVEILAAREKIAAGSLEHHHHHHHH
